# Continuous Blood Glucose Monitoring Reveals Enormous Circadian Variations in Pregnant Diabetic Rats

**DOI:** 10.3389/fendo.2018.00271

**Published:** 2018-05-29

**Authors:** Michaela Golic, Kristin Kräker, Caroline Fischer, Natalia Alenina, Nadine Haase, Florian Herse, Till Schütte, Wolfgang Henrich, Dominik N. Müller, Andreas Busjahn, Michael Bader, Ralf Dechend

**Affiliations:** ^1^Department of Obstetrics, Charité – Universitätsmedizin Berlin, Corporate Member of Freie Universität Berlin, Humboldt-Universität zu Berlin, and Berlin Institute of Health, Berlin, Germany; ^2^Department of Gynecology With Breast Center, Campus Charité Mitte, Charité – Universitätsmedizin Berlin, Corporate Member of Freie Universität Berlin, Humboldt-Universität zu Berlin, and Berlin Institute of Health, Berlin, Germany; ^3^Experimental and Clinical Research Center, a Cooperation Between the Max Delbrück Center for Molecular Medicine in the Helmholtz Association and the Charité – Universitätsmedizin Berlin, Corporate Member of Freie Universität Berlin, Humboldt-Universität zu Berlin, and Berlin Institute of Health, Berlin, Germany; ^4^Berlin Institute of Health (BIH), Berlin, Germany; ^5^Max Delbrück Center for Molecular Medicine in the Helmholtz Association, Berlin, Germany; ^6^Charité – Universitätsmedizin Berlin, Corporate Member of Freie Universität Berlin, Humboldt-Universität zu Berlin, and Berlin Institute of Health, Berlin, Germany; ^7^Partner Site Berlin, Deutsches Zentrum für Herz-Kreislauf-Forschung (DZHK), Berlin, Germany; ^8^Department of Clinical Pharmacology, Goethe-University Hospital Frankfurt, Frankfurt, Germany; ^9^Institute of Pharmacology, Charité – Universitätsmedizin Berlin, Corporate Member of Freie Universität Berlin, Humboldt-Universität zu Berlin, and Berlin Institute of Health, Berlin, Germany; ^10^HealthTwiSt GmbH, Berlin, Germany; ^11^Department of Cardiology and Nephrology, HELIOS Klinikum Berlin, Berlin, Germany

**Keywords:** continuous glucose monitoring, pregnancy, diabetes, rat, circadian variation

## Abstract

**Aim:**

Diabetes in pregnancy is a major burden with acute and long-term consequences. Its treatment requires adequate diagnosis and monitoring of therapy. Many experimental research on diabetes during pregnancy has been performed in rats. Recently, continuous blood glucose monitoring of non-pregnant diabetic rats revealed an increased circadian variability of blood glucose that made a single blood glucose measurement per day inappropriate to reflect glycemic status. Continuous blood glucose measurement has never been performed in pregnant rats. We wanted to perform continuous blood glucose monitoring in pregnant rats to decipher the influence of pregnancy on blood glucose in diabetic and normoglycemic status.

**Methods:**

We used the transgenic Tet29 diabetes rat model with an inducible knock down of the insulin receptor *via* RNA interference upon application of doxycycline (DOX) leading to insulin resistant type II diabetes. All Tet29 rats received a HD-XG telemetry implant (Data Sciences International, USA) that measured blood glucose and activity continuously. Rats were divided into four groups and blood glucose was monitored until end of pregnancy or the corresponding period: Tet29 + DOX (diabetic) non-pregnant, Tet29 + DOX (diabetic) pregnant, Tet29 (normoglycemic) non-pregnant, Tet29 (normoglycemic) pregnant.

**Results:**

All analyzed rats displayed a circadian variation in blood glucose concentration. Circadian variability was much more pronounced in pregnant diabetic rats than in normoglycemic pregnant rats. Pregnancy ameliorated variation in blood glucose in diabetic situation. Pregnancy continuously decreased blood glucose during normoglycemic pregnancy. Diabetic rats were less active than normoglycemic rats. We performed a calculation showing that application of continuous blood glucose measurement reduces animal numbers needed to detect a given effect in experimental setting by decreasing variability and SD.

**Interpretation:**

Continuous blood glucose monitoring *via* a telemetry device in pregnant rats provides a more informative picture of the glycemic situation in comparison to single measurements. This could improve diagnosis and therapy of diabetes, decrease animal numbers within experimental settings, and add another physiological parameter (activity) to the analysis that could be helpful in testing therapeutic concepts targeting blood glucose levels and peripheral muscle function. We propose continuous glucose monitoring as a new tool for the evaluation of pregnant diabetic rats.

## Introduction

Preexisting diabetes during pregnancy and gestational diabetes are a major burden in modern western countries with increasing incidence. It causes not only acute obstetric and neonatal problems (1, 2) but also influences offspring health in later life (3, 4). Correct diagnosis and adequate therapies are required to face diabetes in pregnancy. Many experimental research on diabetes during pregnancy has been performed in rats (5, 6). Although human pregnancy is unique and no animal model provides completely comparable features to human reproduction, the rat offers important similarities to human pregnancy such as hemochorial placentation with deeply into the uterus invading trophoblast cells that mediate uterine spiral artery remodeling (7). This makes the rat an appropriate model to study human pregnancy.

It has long been known that there is a circadian rhythm of insulin secretion and blood glucose concentration in rats (8). Rats are nocturnal animals peaking with their activity, food intake, blood glucose, and insulin secretion at night (9). Recently, it has been shown by continuous blood glucose monitoring that the difference in mean night and mean day blood glucose concentration is less than 10 mg/dL in healthy male rats (10). Interestingly, the circadian variations in blood glucose concentrations are much more pronounced in male diabetic rats with a difference in mean night and mean day blood glucose concentration of more than 140 mg/dL (10). The authors suggested that the perceived outcome of an antidiabetic treatment may depend on the timepoint of glycemic control (10) and since blood glucose is assessed most often in the morning of a working day, primarily for practical reasons, when blood glucose concentration is low, the efficiency of experimental interventions is most likely overestimated in many animal studies (10).

A continuous blood glucose measurement has never been performed in pregnant rats. We wanted to perform continuous blood glucose measurement in pregnant rats to decipher the influence of pregnancy on blood glucose in diabetic and normoglycemic status.

## Materials and Methods

### Tet29 Insulin Receptor Knock Down Rat Model

The animal work has been conducted according to national and international guidelines. The animal study was prospectively approved by the local authorities and the animal research ethics committee of Berlin, Germany (both Landesamt für Gesundheit und Soziales, approval number G 0157/13) and the animal welfare body of the Max Delbrück Center for Molecular Medicine in the Helmholtz Association. We applied the transgenic Tet29 diabetic rat model of insulin resistance (6, 11, 12) (RGD Cat# 2313734, RRID:RGD_2313734), which had been developed on a Sprague-Dawley background by Dr. Michael Bader and his colleagues at the Max Delbrück Center for Molecular Medicine in the Helmholtz Association (11). Animals used in this study were obtained from this breed. The insulin receptor is knocked down *via* RNA interference (induction of small hairpin RNA) upon doxycycline (DOX) administration leading to insulin resistance and hyperglycemia. Without DOX, the Tet construct is silent due to the block of shRNA transcription by the Tet repressor. All rats were kept under standard conditions in cages in the animal house of the Max Delbrück Center for Molecular Medicine in the Helmholtz Association (average 22°C room temperature and 12-/12-h light/dark cycle). The rats had *ad libitum* access to food and drinking water. Rats received an energy reduced diet (9.1 MJ/kg, from ssniff Spezialdiäten GmbH, Soest, Germany) according to the policy of the animal house at the time experiments were performed in 2014 and 2015. The rats were monitored regularly, at least once a day, with evaluation of their physical appearance (e.g., rat fur), behavior, activity, and eating and drinking amounts. Animals were weighed twice a week. At the end of the experiment, all rats and their pups were deeply anesthetized with isoflurane and sacrificed by decapitation.

### Implantation of the HD-XG Glucose Telemetry Transmitter

**Figure 1:**
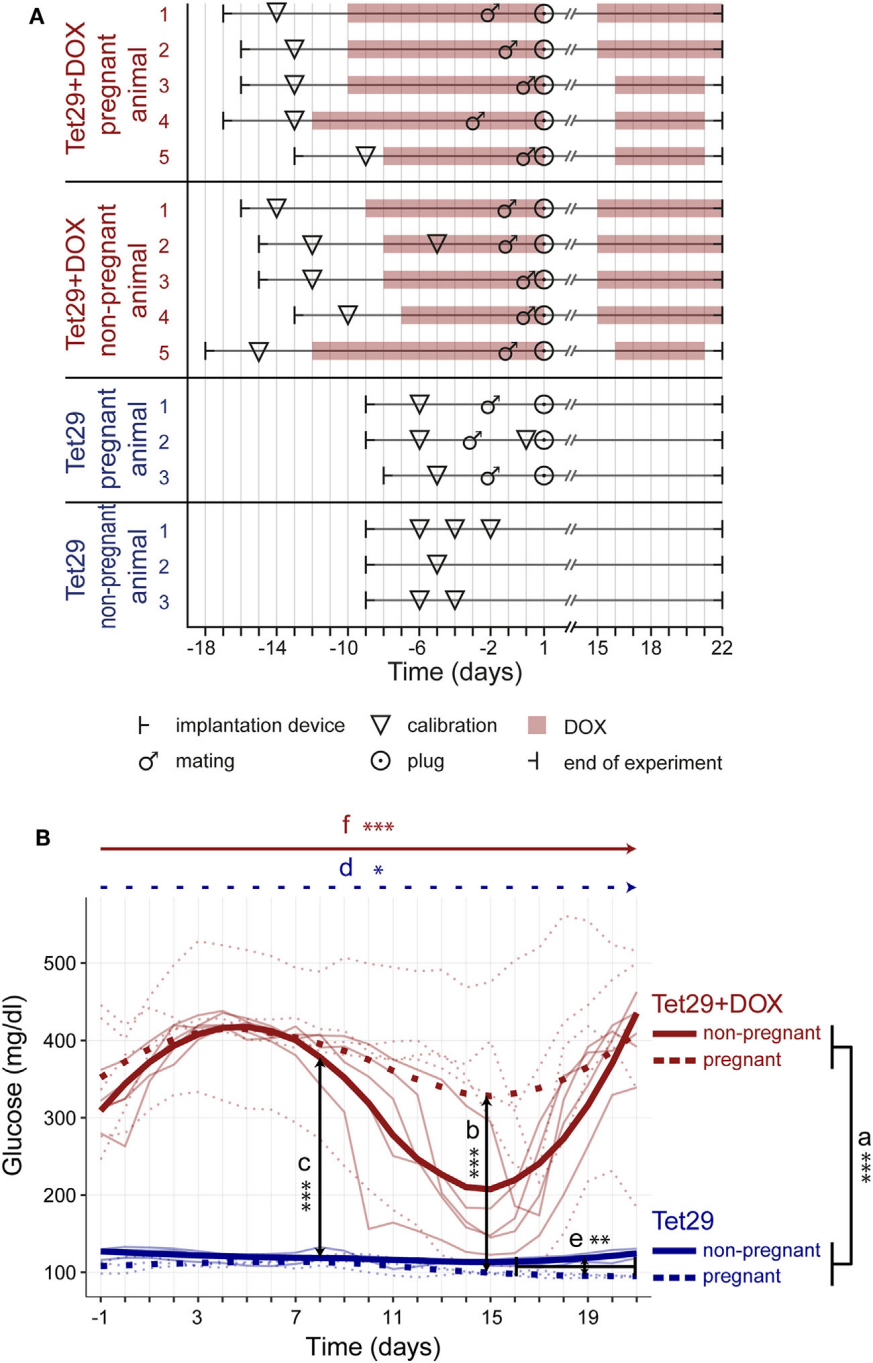
Experimental design and continuous blood glucose monitoring during pregnancy. **(A)** Shown is the experimental design of each rat participating in the study; DOX = Doxycyclin. **(B)** Shown is the mean blood glucose course of each rat per group over time; *n* = 3 non-pregnant Tet29 rats, *n* = 3 pregnant Tet29 rats, *n* = 5 non-pregnant Tet29 + DOX rats, and *n* = 5 pregnant Tet29 + DOX rats. The bold line/symbols show the smoothed averaged course of all animals within a group (LOESS). The days refer to pregnancy or the corresponding period in non-pregnant animals. Tet29 rats were normoglycemic, Tet29 + DOX rats were hyperglycemic. (a) Tet29 + DOX rats, both pregnant and non-pregnant, had higher blood glucose values than Tet29 rats, both pregnant and non-pregnant (repeated measures ANOVA). (b) In addition, pregnant Tet29 + DOX rats had higher blood glucose values than pregnant Tet29 rats (repeated measures ANOVA), and (c) non-pregnant Tet29 + DOX rats had higher blood glucose values than non-pregnant Tet29 rats (repeated measures ANOVA). (d) Blood glucose values decreased during pregnancy in Tet29 rats (ANOVA). (e) In these rats, blood glucose values during late pregnancy were lower than the blood glucose values of non-pregnant Tet29 rats in the corresponding period (*post-hoc* comparison). Pregnancy did not have an influence on mean blood glucose values in diabetic Tet29 + DOX rats (repeated measures ANOVA; *post-hoc* comparison), but there is (f) a significant change in blood glucose values over time in non-pregnant Tet29 + DOX rats (ANOVA).

The HD-XG glucose telemetry transmitter from Data Sciences International (DSI, USA) monitors blood glucose, physical activity, and core body temperature (data not shown). Sixteen female Tet29 rats were used in the following study (Figure 1A). The blood glucose sensor was introduced infrarenally into the abdominal aorta and the transmitter was sutured to the peritoneum. The glucose sensor connector was fixed to the back musculature (left of the vena cava in the region of the iliolumbal veins). The reference electrode was fixed to the inner abdominal wall. The implantation of the glucose telemetry device was performed under isoflurane anesthesia and all efforts were made to minimize suffering. The rats received carprofen 5 mg/kg body weight subcutaneously for analgesia before the surgery and on the day after. Two percent lidocaine gel was applied on the wound at the skin for further analgesia. The wounds were investigated daily to diagnose potential infections and dehiscences immediately.

A two-point calibration of the HD-XG transmitter was performed between 2 and 4 days after implantation of the HD-XG in all rats and in three rats additionally 5, 9, or 10 days after implantation. In one rat, initial calibration had to be repeated 5 and 7 days after implantation of the transmitter. Two-point calibration was conducted by measuring blood glucose concentration manually with the Accu-Chek Aviva device (Roche, Germany) by puncture of the tail vein before and after the intraperitoneal injection of glucose 2–2.7 g/kg body weight and adjusting measurement of the HD-XG device accordingly. After injection of glucose 2–2.7g/kg body weight, the blood glucose increased in average by 247 up to 349 mg/dL (mean based on one calibration result per rat, in the case of several calibrations, the result of the last calibration is used). The calibration was repeated in the case of inappropriate blood glucose increase. After successful initial calibration, the HD-XG device was calibrated at least twice a week with a single blood glucose concentration obtained manually by puncture of the tail vein and adjustment of the HD-XG device accordingly.

### Experimental Design

We investigated four experimental groups: pregnant and non-pregnant Tet29 rats without DOX application (*n* = 3 each), and pregnant and non-pregnant Tet29 + DOX rats with DOX application (*n* = 5 each) making a total of 16 analyzed female rats (Figure 1A). In all rats, experiment started with telemetric device implantation and one or several blood glucose calibrations. All rats were analyzed until the end of the experiment on the morning of pregnancy day 22 (or the respective time point in non-pregnant rats). The presence of a vaginal plug was considered pregnancy day 1. Tet29 + DOX rats received 2 mg/kg body weight DOX several days before conception and mating started when blood glucose ranged between 303 and 388 mg/dL. DOX administration was stopped on pregnancy day 1 and re-started on pregnancy day 15 or 16 until pregnancy day 21 or 22. Pregnant Tet29 rats had 12 pups each, pregnant Tet29 + DOX rats had between 9 and 13 pups. Non-pregnant Tet29 rats were not mated. Non-pregnant Tet29 + DOX rats had been mated and a vaginal plug was visible on pregnancy day 1, but at the time of sacrifice/end of the experiment, their uterus resembled the one of non-pregnant rats (no fetuses, no resorptions, no implantation sites, no other signs of pregnancy). Blood pressure measurement under anesthesia received two pregnant Tet29 + DOX rats on pregnancy day 19, four non-pregnant Tet29 + DOX rats between the respective time point of pregnancy day 17 and 19, and all three pregnant Tet29 rats on pregnancy day 18 or 19 (data not shown).

### Continuous Blood Glucose Measurement and Statistical Analysis

The HD-XG device enabled continuous blood glucose measurement. Blood glucose concentrations in the descending aorta were recorded and analyzed every minute for 23 days (24-h period) in rats maintained in a 12-/12-h light/dark cycle (light on from 6:00 am until 5:59 pm and light off from 6:00 p.m. until 5:59 a.m.). Each rat generated over 29,700 individual blood glucose measurements (median about 32,400) and 529 activity counts (median 552). Activity counts were summed up over 1-h periods.

We focused our statistical analysis on the time period from pregnancy day −1 until pregnancy day 21 (or the respective time period in non-pregnant animals). Pregnancy itself was defined as pregnancy day 1 until pregnancy day 21 (including pregnancy day 21). Time periods were defined by day as early pregnancy (day 1 until day 9), mid pregnancy (day 10 until day 15), and late pregnancy (day 16 until day 21).

The animal experiment was stopped at the morning of pregnancy day 22 at 5:59 a.m. In our statistical analysis, days were defined as the light period between 6:00 a.m. until 5:59 p.m. and nights were defined as the dark period from 6:00 p.m. until 5:59 a.m. Therefore, the statistical analysis included 21 days (24-h periods) of pregnancy. Statistical analysis of non-pregnant Tet29 rats started 8 days after implantation of the device, rather similar to the time point of the pregnant Tet29 rats.

**Figure 2:**
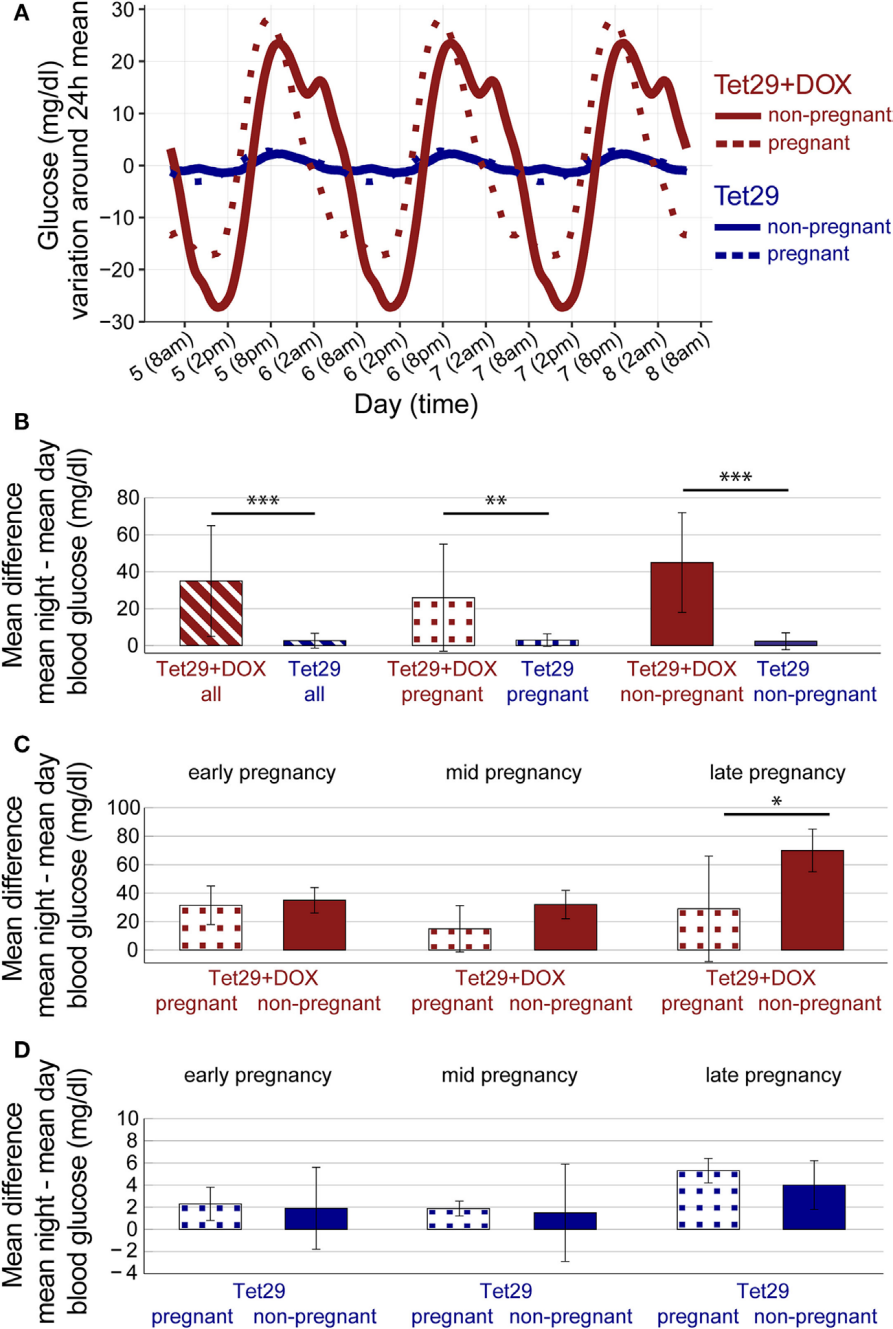
Circadian variation of blood glucose during pregnancy. **(A)** Diurnal variability of blood glucose per group between pregnancy day 5 and 7 or the corresponding period in non-pregnant rats. Data were first averaged over animals within groups for every minute, then smoothed by moving average. DOX = Doxycyclin; There is a circadian variation of blood glucose in all four groups with higher values at night. **(B)** Shown is the mean difference ± SD between mean night and mean day blood glucose during pregnancy or the corresponding period per group. Tet29 + DOX rats, both pregnant and non-pregnant, had a higher difference in night and day blood glucose values than Tet29 rats, both pregnant and non-pregnant (repeated measures ANOVA). In addition, pregnant Tet29 + DOX rats had a higher difference in night and day blood glucose values than pregnant Tet29 rats (repeated measures ANOVA), and non-pregnant Tet29 + DOX rats had a higher difference in night and day blood glucose values than non-pregnant Tet29 rats (repeated measures ANOVA). **(C)** Shown is the mean difference ± SD between mean night and mean day blood glucose during the different periods of pregnancy or the corresponding periods in non-pregnant rats within the Tet29 + DOX rats. Pregnancy decreased the difference between night and day blood glucose in diabetic Tet29 + DOX rats during late pregnancy (*post-hoc* comparison). **(D)** Shown is the mean difference ± SD between mean night and mean day blood glucose during the different periods of pregnancy or the corresponding periods in non-pregnant rats within the Tet29 rats. Pregnancy had no effect on the difference between night and day blood glucose in normoglycemic Tet29 rats during any time period of pregnancy (*post-hoc* comparison). **(A–D)** *n* = 3 non-pregnant Tet29, *n* = 3 pregnant Tet29, *n* = 5 non-pregnant Tet29 + DOX, *n* = 5 pregnant Tet29 + DOX.

**Figure 3:**
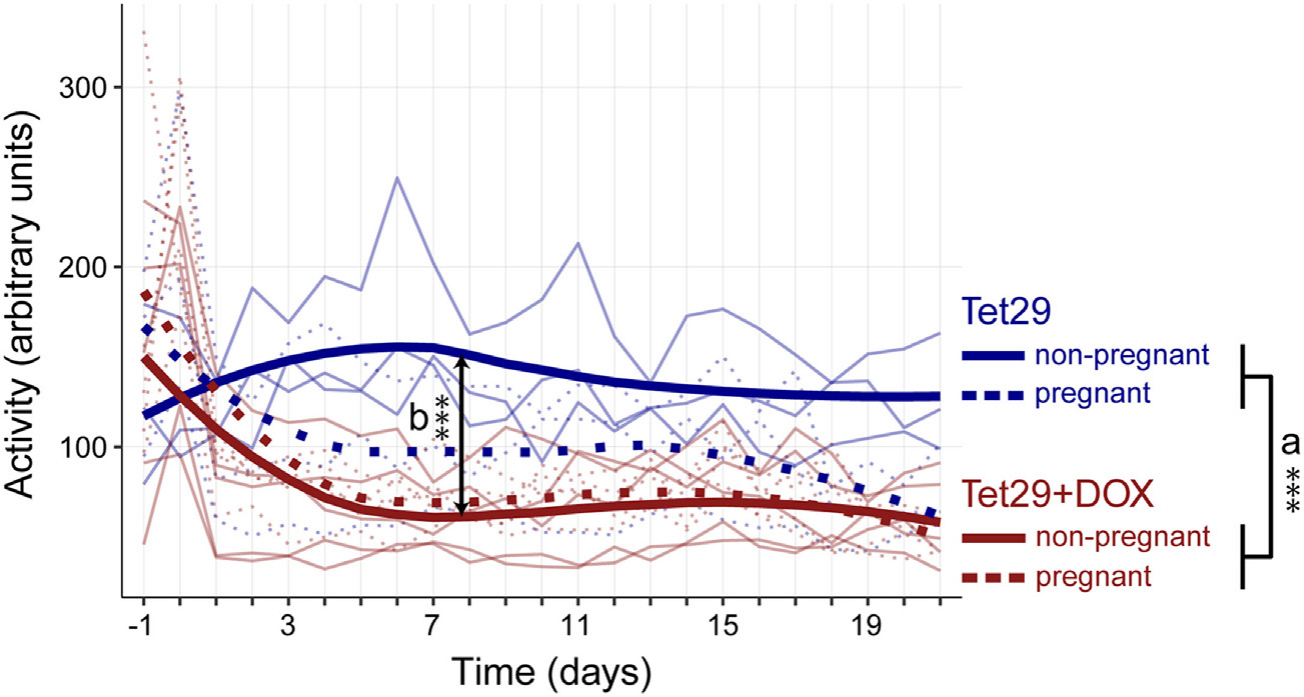
Continuous monitoring of activity during pregnancy. Shown is the mean activity of each rat per group over time. DOX = Doxycyclin; *n* = 3 non-pregnant Tet29 rats, *n* = 3 pregnant Tet29 rats, *n* = 5 non-pregnant Tet29 + DOX rats, and *n* = 5 pregnant Tet29 + DOX rats. The bold line/symbols show the smoothed averaged course of all animals within a group (LOESS). The days refer to pregnancy or the corresponding period in non-pregnant animals. (a) Diabetic Tet29 + DOX rats, both pregnant and non-pregnant, display a lower activity than normoglycemic Tet29 rats, both pregnant and non-pregnant (repeated measures ANOVA). (b) In addition, non-pregnant Tet29 + DOX rats had a lower activity than non-pregnant Tet29 rats (repeated measures ANOVA). There is no difference in activity between pregnant Tet29 + DOX rats and pregnant Tet29 rats (repeated measures ANOVA). Pregnancy did not influence activity in diabetic Tet29 + DOX rats (repeated measures ANOVA), but there was a trend that pregnancy decreased activity in normoglycemic Tet29 rats (repeated measures ANOVA).

Statistical analysis started with an exploratory analysis and the generations of raw data plots (Figure S1 in Supplementary Material). Data were then subjected to a time series analysis and variability was split into the components diurnal rhythm, long-term trend, and remaining random variability (noise; not shown). Outlier detection was based on noise since day/night rhythm would potentially mask extreme values. Extreme values were replaced by the mean of the previous 10 measurements before further analysis. Repeated measures ANOVA, based on daily (24-h period) mean values during pregnancy or the corresponding period in non-pregnant animals, was applied to test the influence of pregnancy on diabetic Tet29 + DOX rats (Figures 1B, 2 and 3) and on normoglycemic Tet29 rats (Figures 2 and 3), and to test the influence of DOX (diabetes) on pregnant rats (Figures 1B, 2B and 3), on non-pregnant rats (Figures 1B, 2B and 3), as well as on all rats (Figures 1B, 2B and 3). *t*-test as *post-hoc* exploratory analysis, based on period average values (*p*-values with adjustment for multiple testing by false discovery rate), was applied to further analyze the influence of pregnancy (early, mid, and late pregnancy) on diabetic Tet29 + DOX rats (Figures 1B and 2C) and on normoglycemic Tet29 rats (Figures 1B and 2D). ANOVA, based on period average values, was performed to test the influence of time in diabetic Tet29 + DOX rats (Figure 1B) and in normoglycemic Tet29 rats (Figure 1B). *P* ≤ 0.05 were considered significant and marked with *. *p* ≤ 0.01 were marked with ** and *p* ≤ 0.001 were marked with ***. Statistical analysis was conducted using R (R Core Team (2017). R: A language and environment for statistical computing. R Foundation for Statistical Computing, Vienna, Austria. https://www.R-project.org/).

## Results

### Continuous Blood Glucose Monitoring During Rat Pregnancy

Tet29 + DOX rats, both pregnant and non-pregnant, had significantly higher blood glucose values than Tet29 rats, both pregnant and non-pregnant (mean blood glucose during pregnancy or the corresponding period in non-pregnant rats of 352 ± 114 vs. 112 ± 12 mg/dL, *p* = 0.001; Figure 1Ba). The blood glucose values initially increased in most Tet29 + DOX rats, followed by a decrease and a second increase. Tet29 rats were normoglycemic with maximum blood glucose values of 133 mg/dL during pregnancy or the corresponding period in non-pregnant Tet29 rats.

When considering pregnant rats only, Tet29 + DOX rats had as well significantly higher blood glucose values than Tet29 rats (mean blood glucose during pregnancy of 377 ± 107 vs. 105 ± 9 mg/dL, *p* = 0.001; Figure 1Bb). In addition, also in non-pregnant status, Tet29 + DOX rats had significantly higher blood glucose values than Tet29 rats (mean blood glucose during the corresponding period of pregnancy of 328 ± 99 vs. 118 ± 7 mg/dL, *p* = 0.001; Figure 1Bc).

### Pregnancy Decreases Blood Glucose Values in Normoglycemic Rats

Blood glucose values decreased continuously during pregnancy in normoglycemic Tet29 rats and were significantly different over time (mean blood glucose of 112 ± 2 mg/dL in early, 104 ± 6 mg/dL in mid, and 96 ± 3 mg/dL in late pregnancy; *p* = 0.030; Figure 1Bd). The mean blood glucose decreased about 16 mg/dL from early to late pregnancy in Tet29 rats. This led to significantly lower blood glucose values during late pregnancy in comparison to the blood glucose values of non-pregnant Tet29 rats in the corresponding period (96 ± 3 vs. 117 ± 6 mg/dL; *p* = 0.004; Figure 1Be). Non-pregnant Tet29 rats did not display such a prominent change in blood glucose values over time (mean blood glucose of 121 ± 6 mg/dL, 114 ± 3 mg/dL, and 117 ± 6 mg/dL in the corresponding period to early, mid, and late pregnancy, respectively, *p* = 0.372; Figure 1B). The blood glucose values of Tet29 rats during early and mid pregnancy were not significantly different in comparison to the blood glucose values of non-pregnant Tet29 rats in the corresponding period (*p* = 0.102 and *p* = 0.072, respectively; Figure 1B).

### Pregnancy Decreases Variation in Blood Glucose Values in Diabetic Rats

Pregnancy did not have a significant influence on mean blood glucose values in diabetic rats (*p* = 0.315; Figure 1B). The blood glucose values of Tet29 + DOX rats during early, mid, and late pregnancy were not significantly different to blood glucose values of non-pregnant Tet29 + DOX rats in the corresponding period (mean blood glucose of 407 ± 71 mg/dL in early, 350 ± 123 mg/dL in mid, and 357 ± 133 mg/dL in late pregnancy vs. 399 ± 12 mg/dL (*p* = 0.808), 245 ± 72 mg/dL (*p* = 0.140), and 306 ± 42 mg/dL (*p* = 0.451) in the corresponding period to early, mid, and late pregnancy). But when focusing on the blood glucose course within each group, the non-pregnant Tet29 + DOX rats had a significant change in blood glucose values over time (*p* = 0.001; Figure 1Bf). There was a decrease in blood glucose of 154 mg/dL from the early to the mid period followed by an increase of blood glucose of 61 mg/dL from the mid to the late period in non-pregnant Tet29 + DOX rats. Pregnant Tet29 + DOX rats did not display such a prominent change in blood glucose values over time (*p* = 0.121; Figure 1B). Blood glucose decreased about 57 mg/dL from early to mid pregnancy and increased about 7 mg/dL from mid to late pregnancy in pregnant Tet29 rats.

### Diabetes Increases Circadian Variation in Blood Glucose in Pregnant Rats

There was a circadian variation of blood glucose in all rats, independent of pregnancy and diabetic status (Figure 2A). Blood glucose was highest around 8 p.m. (2 h after start of dark cycle) and lowest around 2 p.m. (8 h after initiation of light cycle) within 24 h (Figure 2A). There was a continuous decrease in blood glucose over 18 h from 8 to 2 p.m., followed by a faster (over 6 h), but still continuous increase in blood glucose from 2 until 8 p.m. (Figure 2A). Diabetic Tet29 + DOX rats, both pregnant and non-pregnant, had a significantly higher difference in night and day blood glucose values than normoglycemic Tet29 rats, both pregnant and non-pregnant (mean difference between mean night and mean day blood glucose during pregnancy or the corresponding period in non-pregnant rats of 35 ± 30 vs. 2.7 ± 4.0 mg/dL, *p* = 0.001; Figure 2B).

When considering pregnant rats only, diabetic Tet29 + DOX rats had as well a significantly higher difference in night and day blood glucose values than normoglycemic Tet29 rats (mean difference between mean night and mean day blood glucose during pregnancy of 26 ± 29 vs. 3.0 ± 3.4 mg/dL, *p* = 0.006; Figure 2B). In addition, also in non-pregnant status, diabetic Tet29 + DOX rats had a significantly higher difference in night and day blood glucose values than normoglycemic Tet29 rats (mean difference between mean night and mean day blood glucose during the corresponding period of pregnancy of 45 ± 27 vs. 2.4 ± 4.6 mg/dL, *p* = 0.001; Figure 2B).

### Pregnancy Decreases Circadian Variation in Blood Glucose in Diabetic Rats

Pregnancy significantly decreased the mean difference between mean night and mean day blood glucose in diabetic Tet29 + DOX rats (*p* = 0.007; repeated measures ANOVA). The mean difference between mean night and mean day blood glucose during late pregnancy was significantly smaller than the one of non-pregnant Tet29 + DOX rats during the corresponding period (29 ± 37 vs. 70 ± 15 mg/dL; *p* = 0.049; Figure 2C). There was no significant change in mean difference between mean night and mean day blood glucose between pregnant and non-pregnant Tet29 + DOX rats during early and mid pregnancy or the corresponding period (early pregnancy 31.5 ± 13.5 vs. corresponding period 35 ± 9 mg/dL, *p* = 0.655; mid pregnancy 15.0 ± 16.3 vs. corresponding period 32 ± 10 mg/dL, *p* = 0.079; Figure 2C).

Pregnancy did not significantly influence mean difference between mean night and mean day blood glucose in normoglycemic Tet29 rats (*p* = 0.732; repeated measures ANOVA). The mean difference between mean night and mean day blood glucose values was 2.3 ± 1.5 mg/dL in early, 1.89 ± 0.67 mg/dL in mid, and 5.3 ± 1.1 mg/dL in late pregnancy in normoglycemic Tet29 rats in comparison to 1.9 ± 3.7 mg/dL (*p* = 0.852), 1.5 ± 4.4 mg/dL (*p* = 0.900), and 4.0 ± 2.2 mg/dL (*p* = 0.434) in non-pregnant Tet29 rats in the corresponding periods (Figure 2D).

### Diabetes Reduces Activity in Rats

Besides continuous blood glucose monitoring, the telemetric device enabled also monitoring of activity (Figure 3). Diabetic Tet29 + DOX rats, both pregnant and non-pregnant, had a significantly lower activity than normoglycemic Tet29 rats, both pregnant and non-pregnant (mean activity during pregnancy or the corresponding period in non-pregnant rats of 69 ± 57 vs. 115 ± 95 arbitrary units, *p* = 0.001; Figure 3a).

When considering pregnant rats only, there was no significant difference in activity between diabetic Tet29 + DOX rats and normoglycemic Tet29 rats (mean activity during pregnancy of 72 ± 17 vs. 92 ± 34 arbitrary units, *p* = 0.215, Figure 3). In non-pregnant status, Tet29 + DOX rats had significantly lower activity than normoglycemic Tet29 rats (mean activity during the corresponding period of pregnancy of 67 ± 25 vs. 139 ± 32 arbitrary units, *p* = 0.001; Figure 3b). Pregnancy neither significantly influenced the activity in diabetic Tet29 + DOX rats (*p* = 0.680; Figure 3) nor in normoglycemic Tet29 rats (*p* = 0.084; Figure 3), but there was a trend to lower activity in pregnant Tet29 rats in comparison to non-pregnant Tet29 rats.

### Continuous Blood Glucose Monitoring Reduces Sample Size

**Figure 4:**
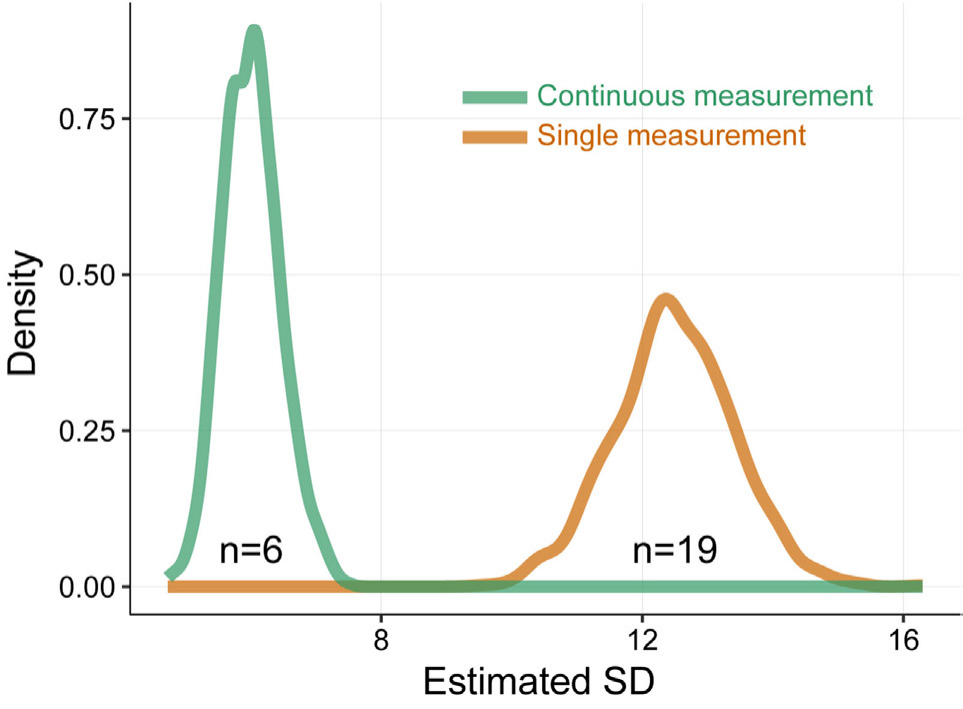
Simulation on sample size requirements. Simulation to evaluate the effect of continuous blood glucose measurement on sample size requirements. The simulation is based on 1,000 simulation runs with 100 animals per run. SD of continuous blood glucose measurements and single measurements is computed and aggregated over simulations. The sample size necessary to detect a 10% mean change with 80% power is computed based on average SD. The single measurement-based analysis overestimates the within-sample variability. With a “true” SD of 6, the single measurement-based analysis estimates an SD of 12.5, whereas the continuous blood glucose measurement-based analysis estimates an SD of 6.0. This results in larger sample sizes required to detect a given effect in single measurement-based analysis (19 vs. 6 in continuous blood glucose measurement-based analysis).

Finally, we evaluated the effect of continuous blood glucose measurement on sample size requirements (Figure 4). Based on the experience of our analysis, we simulated a scenario to calculate the sample size necessary to detect a 10% mean change with 80% power based on average SD. Continuous blood glucose measurement provides a lower variability and thus lower SD in measured values than single measurement (SD of 6.0 vs. SD of 12.5 within single measurement), which results in smaller sample sizes required to detect a given effect (*n* = 6 vs. *n* = 19 within a single measurement analysis).

## Discussion

We successfully employed the method of continuous blood glucose and activity measurement during rat pregnancy in normoglycemic and diabetic status. We showed that (1) pregnant diabetic rats display a much more pronounced circadian variation in blood glucose than normoglycemic pregnant rats, (2) pregnancy ameliorates variation in blood glucose in diabetic status, and (3) pregnancy continuously decreases blood glucose during normoglycemic pregnancy. In addition, we showed that (4) diabetes reduces activity in non-pregnant rats, and we (5) simulated that application of continuous blood glucose measurement could reduce sample size. To the best of our knowledge, this has not been described before.

It has already been shown by other authors that diabetes increases circadian variability of blood glucose in non-pregnant rats (10). We provide data that this phenomenon applies for pregnant rats as well. The difference between night and day blood glucose was not just absolutely higher in diabetic pregnant rats in comparison to normoglycemic pregnant rats, it is also relatively higher with regard to the mean blood glucose during pregnancy (6.9 vs. 2.9%). Interestingly, pregnancy reduced the circadian variation in blood glucose and the variation in blood glucose within time in the diabetic rats of our study. We are not aware of literature about this phenomenon, but propose future studies to further elucidate blood glucose variation during diabetic pregnancy. After all, it is not fully understood to which extent high blood glucose during diabetic pregnancy and the variation in blood glucose within diabetic pregnancy harms the fetus. We interpret our data of reduced glucose variation during diabetic pregnancy as a protective mechanism to supply the fetus with continuous blood glucose. One could speculate that this protective mechanism might be reduced or missing in diabetic pregnancies with very severe fetal outcome.

Although pregnancy reduced circadian variation in blood glucose and the variation in blood glucose with time in the diabetic rats of our study, we did not see an influence of pregnancy on mean blood glucose values. Owing to our rat model, we had to apply DOX to induce diabetes. DOX application was stopped on pregnancy day 1 (or the corresponding period in non-pregnant animals) to reduce embryo-/fetotoxicity and to avoid a further increase in blood glucose. DOX was re-started on pregnancy day 15 or 16 (or the corresponding period in non-pregnant animals). The blood glucose values of most of the analyzed diabetic rats mirror this DOX protocol with an initial increase of blood glucose, followed by a decrease and a second increase of blood glucose values. Thus, we cannot conclude that the blood glucose course we analyzed is the typical one of diabetic pregnant rats. It might well be that DOX application had masked an increase or decrease of blood glucose during “natural” diabetic rat pregnancy. But we were able to analyze the influence of pregnancy in our rat diabetes model by comparison with non-pregnant diabetic rats, which were also treated with DOX, to reveal the difference in variation in blood glucose values mentioned above.

Although human pregnancy induces peripheral insulin resistance (13), most women display normal glucose tolerance during pregnancy due to increased insulin secretion (13). We are not aware of any studies on physiological blood glucose courses during normoglycemic pregnancy in humans or rodents. Our study revealed a decrease in blood glucose throughout normoglycemic rat pregnancy. The low blood glucose in the rat at late pregnancy might be the result of the increasing nutritional demand of the fetus possibly triggered by increased maternal insulin secretion. We cannot make any statements about insulin secretion in the normoglycemic pregnant rats of our study and thus propose future studies on physiological insulin secretion and insulin resistance during rat pregnancy.

Interestingly, our study also revealed that diabetes enormously reduces activity in non-pregnant rats. Diabetes is often considered a consequence of sedentary lifestyle. Our data propose that it could work reversely as well. The association between insulin resistance/diabetes and reduced activity is not fully understood so far. Insulin resistance might lead to decreased glucose uptake of skeletal muscle decreasing its function. During heart failure, which is often accompanied by insulin resistance, metabolic failure of the myocardium and of peripheral tissues has been described in Ref. (14). Rat activity can be used as a parameter for skeletal muscle function and energy consumption which are of relevance when analyzing blood glucose and insulin metabolism. In addition, the HD-XG device could be a valuable tool to test therapeutic concepts targeting blood glucose levels and peripheral muscle function (activity). Our data show that pregnancy does not influence activity in diabetic status, which might be because diabetic rats generally move less and a further decrease due to pregnancy would not be relevant. Pregnancy also did not influence activity in normoglycemic rats of our study, but there was a clear trend toward less activity during pregnancy which might not have become significant due to the low animal numbers used (*n* = 3 vs. *n* = 3).

The circadian variation leads to a significant change in blood glucose making a random single blood glucose value within an experiment an inappropriate parameter to analyze the glycemic status of a rat, especially when performed at different time points during the day. This problem aggravates when evaluating diabetic rats. Random fluctuation further increases within-animal variation in blood glucose in diabetic rats. We thus propose continuous blood glucose measurement *via* telemetric device in pregnant and non-pregnant diabetic rats to improve data quality. In theory, repetitive single blood glucose measurements could also provide continuous blood glucose monitoring, but they are impractical to conduct and accompanied by stress for the animal, which would confound the results. Importantly, besides improvement of data quality, continuous blood glucose monitoring could reduce the number of required samples to detect a given effect. There would be a reduction of more than 68% of animals needed in our calculated scenario. This supports the three Rs (Replace, Reduce, and Refine), the guiding principles underlying animal use in scientific research.

### Limitations of the Study

Limitations of our study are the low animal numbers we used and the partly different treatment between the animal groups (e.g., different timepoint and number of calibrations, different total DOX dosages, and blood pressure measurement under short-time anesthesia in some animals). Another limitation is that Data Sciences International (DSI, USA) guarantees 30 days of continuous blood glucose measurement with their HD-XG glucose telemetry transmitter, but we used the transmitter between 31 and 41 days due to the long study protocol with pregnancy itself already lasting 22 days. Rats should recover at least 5 days after implantation of the glucose monitoring device before any manipulation like mating can start which then sometimes takes some days until successful conception happens. This makes it difficult to perform our analysis in normoglycemic rats within 30 days, and it is even impossible to perform it in the diabetic rats, which need to become diabetic *via* DOX application for several days before mating. Although our raw data do not exhibit signs of device failure and the end of the warranty does not mean that the device stops functioning correctly, we cannot exclude any kind of device failure at the end of our study or differences between the groups in dependence of the duration of the device. Additional limitations are that we did not mate the non-pregnant animals with sterile males and that the diabetic animals we considered non-pregnant may have been briefly pregnant. These rats had a vaginal plug, a sign for copulation that is often used as a marker of pregnancy in healthy rats. In the diabetic rats of our model, however, fertility is decreased (data not shown), and pregnancy rates after vaginal plug are lower than in healthy rats. It is very likely that the diabetic Tet29 rats we consider non-pregnant were never pregnant; however, we cannot exclude that they were pregnant for a couple of days before experiencing a very early pregnancy loss. Because of the limitations mentioned, our results need to be replicated in studies with larger sample size and different animal models.

## Conclusion

Taken together, continuous blood glucose monitoring *via* telemetry device in pregnant and non-pregnant rats provides a more informative picture of the glycemic situation of an individual that could improve diagnosis and therapy of diabetes mellitus, decrease experimental animal numbers and add another physiological parameter (activity) to the analysis.

## Data Availability Statement

The manuscript provides the data generated by the authors supporting the conclusion of the manuscript. The raw data will be made available by the authors, without undue reservation, to any qualified researcher.

## Ethics Statement

The animal work has been conducted according to national and international guidelines. The animal study was prospectively approved by the local authorities and the animal research ethics committee of Berlin, Germany (both Landesamt für Gesundheit und Soziales, approval number G 0157/13) and the animal welfare body of the Max Delbrück Center for Molecular Medicine in the Helmholtz Association.

## Author Contributions

MG, NA, MB, and RD contributed conception and design of the study. AB performed the statistical analysis. MG, CF, and AB analyzed the data. All authors substantially contributed to the interpretation of the data. MG and RD drafted the manuscript. All authors revised the manuscript critically for important intellectual content. All authors provided approval for publication of the content and agreed to be accountable for all aspects of the work.

## Conflict of Interest Statement

Data Sciences International (DSI, St. Paul, MN, USA) donated the telemetric devices and paid Dr. Andreas Busjahn for the statistical analysis.

## References

[1] Yang X Hsu-Hage B Zhang H Zhang C Zhang Y Zhang C. Women with impaired glucose tolerance during pregnancy have significantly poor pregnancy outcomes. Diabetes Care (2002) 25(9):1619–24. 10.2337/diacare.25.9.1619 12196437

[2] Landon MB Spong CY Thom E Carpenter MW Ramin SM Casey B A multicenter, randomized trial of treatment for mild gestational diabetes. N Engl J Med (2009) 361(14):1339–48. 10.1056/NEJMoa0902430 19797280 PMC2804874

[3] Dabelea D Hanson RL Lindsay RS Pettitt DJ Imperatore G Gabir MM Intrauterine exposure to diabetes conveys risks for type 2 diabetes and obesity: a study of discordant sibships. Diabetes (2000) 49(12):2208–11. 10.2337/diabetes.49.12.2208 11118027

[4] Hillier TA Pedula KL Schmidt MM Mullen JA Charles MA Pettitt DJ. Childhood obesity and metabolic imprinting: the ongoing effects of maternal hyperglycemia. Diabetes Care (2007) 30(9):2287–92. 10.2337/dc06-2361 17519427

[5] Canavan JP Goldspink DF. Maternal diabetes in rats. II. Effects on fetal growth and protein turnover. Diabetes (1988) 37(12):1671–7. 10.2337/diabetes.37.12.1671 2461324

[6] Golic M Stojanovska V Bendix I Wehner A Herse F Haase N Diabetes mellitus in pregnancy leads to growth restriction and epigenetic modification of the Srebf2 gene in rat fetuses. Hypertension (2018) 71(5):911–20. 10.1161/HYPERTENSIONAHA.117.10782 29610268

[7] Soares MJ Chakraborty D Karim Rumi MA Konno T Renaud SJ. Rat placentation: an experimental model for investigating the hemochorial maternal-fetal interface. Placenta (2012) 33(4):233–43. 10.1016/j.placenta.2011.11.026 22284666 PMC3288880

[8] Kalsbeek A Strubbe JH. Circadian control of insulin secretion is independent of the temporal distribution of feeding. Physiol Behav (1998) 63(4):553–8. 10.1016/S0031-9384(97)00493-9 9523898

[9] Qian J Block GD Colwell CS Matveyenko AV. Consequences of exposure to light at night on the pancreatic islet circadian clock and function in rats. Diabetes (2013) 62(10):3469–78. 10.2337/db12-1543 23775768 PMC3781472

[10] King AJ Austin AL Nandi M Bowe JE Diabetes in rats is cured by islet transplantation…but only during daytime. Cell Transplant (2017) 26(1):171–2. 10.3727/096368916X692258 27502050 PMC5657683

[11] Kotnik K Popova E Todiras M Mori MA Alenina N Seibler J Inducible transgenic rat model for diabetes mellitus based on shRNA-mediated gene knockdown. PLoS One (2009) 4(4):e5124. 10.1371/journal.pone.0005124 19340286 PMC2659743

[12] Santos SH Giani JF Burghi V Miquet JG Qadri F Braga JF Oral administration of angiotensin-(1-7) ameliorates type 2 diabetes in rats. J Mol Med (Berl) (2014) 92(3):255–65. 10.1007/s00109-013-1087-0 24162089

[13] Kühl C. Insulin secretion and insulin resistance in pregnancy and GDM. Implications for diagnosis and management. Diabetes (1991) 40(Suppl 2):18–24. 10.2337/diab.40.2.S18 1748255

[14] Doehner W Frenneaux M Anker SD. Metabolic impairment in heart failure: the myocardial and systemic perspective. J Am Coll Cardiol (2014) 64(13):1388–400. 10.1016/j.jacc.2014.04.083 25257642

