# Supplementary material for: Continuous Blood Glucose Monitoring Reveals Enormous Circadian Variations in Pregnant Diabetic Rats
Source: Front Endocrinol (Lausanne). 2018 May 29;9:271. doi: 10.3389/fendo.2018.00271 (PMC5986873; doi:10.3389/fendo.2018.00271)
Supplement: Supplementary file 1 [file Image_1.pdf]

Supplementary Material

Continuous Blood Glucose Monitoring Reveals Enormous Circadian Variations in Pregnant Diabetic Rats

Michaela Golic, Kristin Kräker, Caroline Fischer, Natalia Alenina, Nadine Haase, Florian Herse, Till Schütte, Wolfgang Henrich, Dominik N Müller, Andreas Busjahn, Michael Bader, Ralf Dechend*

* **Correspondence:** Corresponding author ralf.dechend@charite.de

Supplementary Figure S1:

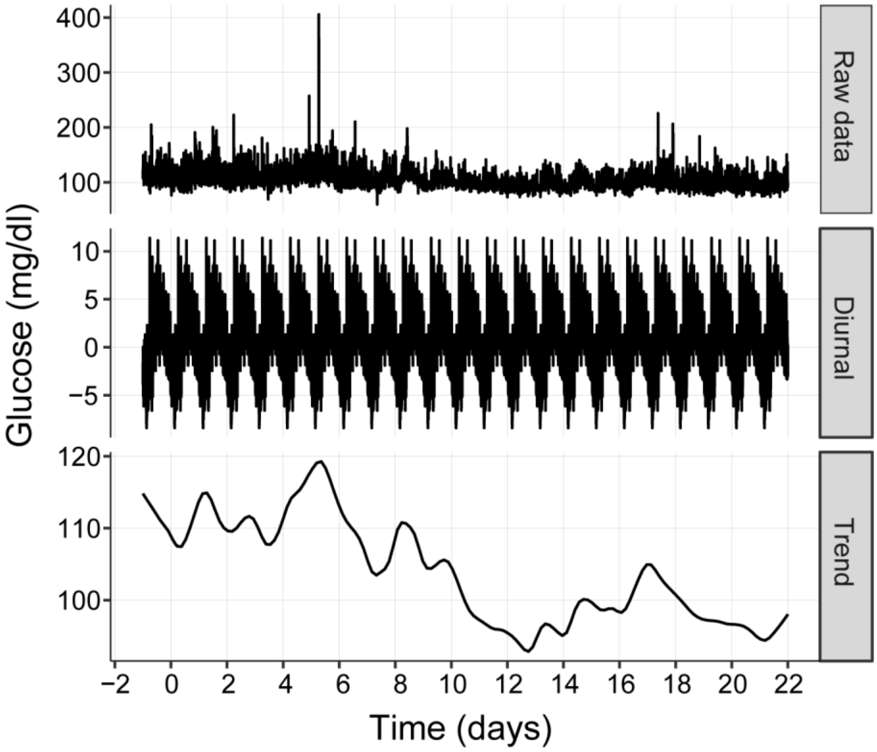

Supplementary Figure 1. Analysis of continuous blood glucose monitoring during pregnancy. Representative blood glucose analysis of a normoglycaemic Tet29 pregnant rat (rat number 1 according to Figure 1A). Shown are measured raw data (first line) and the diurnal curve (second line), which displays the constant averaged rhythm of the whole analysis. The trend curve in blood glucose (third line) is an extreme smoothing of the data that results after subtraction of diurnal data from raw data. During pregnancy, the mean blood glucose was 103±11 mg/dl, the highest blood glucose value was 118 mg/dl (pregnancy day 5), and the lowest blood glucose value 93 mg/dl (pregnancy day 12). There was a trend of blood glucose decrease during the time of pregnancy.
